# Socioecological Predictors of Child Flourishing and Family Resilience Status Among Children with Adverse Childhood Experiences

**DOI:** 10.3390/ijerph23030277

**Published:** 2026-02-24

**Authors:** Eunice Lee

**Affiliations:** School of Social Work, College of Health, Cleveland State University, Cleveland, OH 44115, USA; e.lee85@csuohio.edu

**Keywords:** socioecological predictors, child flourishing, resilience, adverse childhood experiences

## Abstract

**Highlights:**

**Public health relevance—How does this work relate to a public health issue?**

- Identifies how adverse childhood experiences (ACEs), one of the major public health concerns, intersect with child flourishing and family resilience, shifting attention from deficits to positive adaptation.
- Examines multilevel socioecological factors influencing resilience among children with ACEs, aligning with public health priorities on social and environmental determinants of health.

**Public health significance—Why is this work of significance to public health?**

- Demonstrates that nearly half of children with ACEs show both child flourishing and family resilience, while a substantial minority exhibit neither, highlighting meaningful heterogeneity in resilience within a high-risk population.
- Provides evidence that school safety, supportive neighborhoods, and neighborhood amenities are positively associated with resilient status, whereas parenting stress and higher cumulative ACE exposure are negatively associated, highlighting modifiable contextual factors that can be targeted in public health efforts.

**Public health implications—What are the key implications or messages for practitioners, policy makers and/or researchers in public health?**

- Suggests that promoting resilience among children with ACEs requires coordinated, multi-level strategies to reduce parenting stress and to strengthen safe school, supportive neighborhoods, and access to neighborhood amenities.
- Underscores the need for public health policies and programs that move beyond ACE screening alone to invest in upstream, context-focused interventions that enable children and families not only to avoid negative outcomes, but to flourish.

**Abstract:**

Objective: The objective of this study is to identify socioecological predictors of child flourishing and family resilience status among U.S. school-aged children with a history of childhood adverse experiences (ACEs). Study design: This study is a secondary analysis of the 2024 National Survey of Children’s Health (NSCH), a cross-sectional and nationally representative survey of noninstitutionalized U.S. children. Parental reports of child flourishing and family resilience measures were used to create a categorical variable with four different types of resilience status: neither child flourishing nor family resilience, child flourishing only, family resilience only, and both child flourishing and family resilience. Multinomial logistic regression was performed to investigate multi-level factors predicting the child flourishing and family resilience status of children aged between 6 and 17 years (N = 13,571). Results: Among children with ACEs, 46.5% were classified as both child flourishing and family resilience; 33.3% were in the family resilience only group, 14.0% were in the neither child flourishing nor family resilience group, and 6.6% were in the child flourishing only group. School safety, supportive neighborhoods, and neighborhood amenities were positively associated with resilient status, whereas parenting stress and higher cumulative ACE exposure were negatively associated with the resilience status. Conclusions: Children and families can demonstrate resilience despite ACE exposure in the presence of supportive socioecological conditions. Efforts to promote healthy development among children with ACEs may benefit from multilevel prevention and intervention strategies that strengthen caregiving resources, reduce parenting stress, and improve school and neighborhood environments.

## 1. Introduction

Adverse childhood experiences (ACEs) are traumatic and stressful events spanning from child maltreatment to environmental stressors, including economic hardship, household challenges, parental substance abuse, mental illness, racial discrimination, and community violence [1,2,3,4,5]. Substantial research describes ACEs and their harmful impact on the health and wellbeing in childhood and throughout the life course. Specifically, ACEs have been linked to a wide range of negative outcomes, including but not limited to depression [6,7], substance use disorder [8,9], suicidal behaviors [10], poor school readiness [11], low school engagement [12], poor academic performance [13,14], and health risk behaviors [15,16]. In the long-term, the toxic stress resulting from severe and chronic adversities during childhood may increase the risk of chronic diseases and premature death in later adulthood [4,17,18,19,20,21].

A substantial body of evidence further demonstrates a dose–response relationship in which the likelihood of adverse health and well-being outcomes increases as the number of ACEs increases. For example, Felitti and colleagues [1] reported a strong graded relationship between cumulative exposure to childhood abuse and household challenges and multiple adult risk factors and leading causes of death, suggesting that ACEs exert additive effects rather than reflecting a simple exposed versus unexposed difference. A systematic review and meta-analysis similarly found that individuals with four or more ACEs had substantially elevated risk across a wide range of outcomes compared with those with none, reinforcing evidence that ACE exposure operates cumulatively across the life course [22]. The Center for Disease Control and Prevention (CDC) also summarizes this evidence as a graded dose–response relationship, emphasizing that as ACE counts rise, the likelihood of negative health and social outcomes increases. For instance, analyses using the Youth Risk Behavior Survey have shown particularly strong associations for four or more ACEs with suicidal ideation, suicide attempts, and current prescription opioid misuse [23].

Not all children who have ACEs develop negative outcomes. In fact, many children who are exposed to adversities fare well despite early hardships. This successful adaptation in the face of significant challenges is identified as resilience [24,25,26,27,28]. Increasingly, resilience is understood not as a fixed trait or a single outcome, but as a multi-level, dynamic process that emerges through ongoing interactions between children and the social and environmental contexts in which they live [26]. This perspective highlights the importance of distinguishing child-level adaptation from family-level adaptation, rather than treating resilience as a single, undifferentiated indicator following adversity. Conceptually and practically, distinguishing these domains helps capture heterogeneity in adaptation and clarifies intervention targets. For example, the National Survey of Children’s Health (NSCH) operationalizes child flourishing and family resilience as related but conceptually distinct processes [27]. In the NSCH, child flourishing reflects children’s positive functioning and thriving, assessed through indicators such as curiosity, persistence, and self-regulation (e.g., staying calm) among school-aged children. In contrast, family resilience captures a core family coping process—how families respond to and manage problems—measured by whether families talk together about what to do, work together to solve problems, maintain hope, and draw on family strengths [27].

Importantly, these domains may align, but they can also diverge. A family may demonstrate strong collective coping and problem-solving while a child continues to struggle with daily functioning or emotional regulation. Conversely, a child may appear to thrive despite limited family-level adaptive capacity. Accordingly, relying on a single resilience measure, either child-focused or family-focused, may not capture meaningful heterogeneity in adaptation following ACE exposure, particularly when strengths in one domain coexist with vulnerabilities in the other. Practically, this can misdirect intervention strategies. Supports that strengthen family communication and collaborative coping may not fully address a child’s self-regulation or engagement, while child-focused supports may be insufficient if ongoing family stressors undermine sustained wellbeing [29,30,31].

Additionally, prior research on resilience among children exposed to ACEs has largely emphasized individual- (e.g., social orientation, self-regulation, self-esteem) and family- (e.g., responsive parents, parenting stress, and family cohesion) level characteristics [32,33,34,35]. Particularly, a stress process perspective suggests that adversity increases family strain, which can shape children’s adjustment through its effects on caregivers’ emotional resources and parenting practices [36]. Parenting stress is a particularly relevant indicator of this pathway because it reflects caregivers’ perceived strain in managing parenting demands. Elevated parenting stress has been linked to less consistent and less responsive parenting, reduced emotional availability, and greater family conflict, each of which is associated with poorer child socioemotional and behavioral outcomes and may constrain children’s capacity to thrive following adversity [37].

While family-level stress processes are central, an ecological view of resilience emphasizes that children’s adaptation to adversity is also shaped by contexts outside the home. However, compared with studies focusing on individual and family characteristics, fewer studies have examined how school and neighborhood contexts relate to resilience outcomes among children with adversity exposure. Emerging evidence suggests that supportive environments can buffer adversity by providing safety, stability, and access to social resources [38,39]. In schools, a positive climate characterized by providing safety and connectedness has been linked to better mental health and positive functioning [40]. Neighborhood social cohesion and supportive community environments have been associated with better child wellbeing and higher likelihood of positive functioning [41,42]. Related work also indicates that safe neighborhoods and supportive communities may protect children from the health effects of adversity [43]. In contrast, distracting neighborhood conditions and physical disorder can signal threat and chronic stress, constrain outdoor plans and social interactions, and contribute to caregiver strain, which may undermine children’s socioemotional and behavioral adjustment [44]. Given the developmental period, these school and neighborhood conditions may be especially imperative among school-aged children, who spend significant time in school and increasingly interact with peers and community settings outside the home.

Together, despite the importance of this multi-level perspective, limited research simultaneously considers factors across multiple levels of the social ecology while also considering child flourishing and family resilience as related but distinct domains. This gap underscores the need for more comprehensive studies among children who have experienced adversity [30]. Accordingly, the current study examined multilevel socioecological predictors, including parenting stress and neighborhood-related characteristics, to identify key influences on four types of child flourishing and family resilience status among a nationally representative sample of U.S. school-aged children (6–17 years) with a history of ACE exposure.

## 2. Methods

### 2.1. Data and Sample

The current study used data from the 2024 National Survey of Children’s Health (NSCH). The 2024 NSCH included 51,375 noninstitutionalized children between less than 1 year and 17 years of age at the time of sampling [45]. The NSCH aimed to collect information on the health, health care, and well-being of children as well as their family and broad environmental factors. Between June 2024 and February 2025, the data were collected from parents or guardians (hereafter, parents) who knew enough about their randomly chosen child for the study. The survey was conducted both in English and Spanish. The overall response rate in the 2024 survey was 36.3% [45].

**Figure 1:**
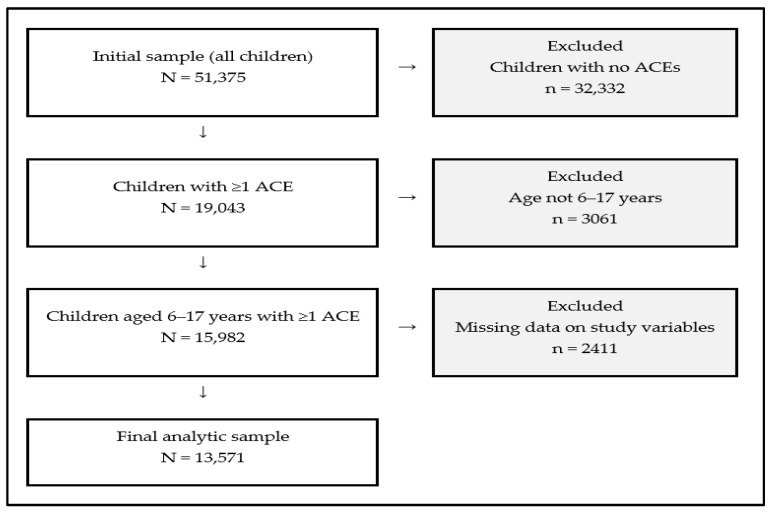
Final Analytic Sample Selection Flow Diagram.

As shown in Figure 1, the analytic sample was restricted to children with at least one ACE exposure, which excluded 32,332 children with no reported ACEs. The sample was further limited to school-aged children (6–17 years) because the child flourishing measure is only available for this age group, excluding 3061 children younger than 6 years. Item-level missingness was low across key study variables (child flourishing = 0.3%, family resilience = 0.9%, parenting stress = 0.3%, school safety = 1.1%, neighborhood safety = 0.7%, supportive neighborhood = 0.7%, neighborhood amenities = 0.8%, and ACE items = 0.3–1.6%). In total, 2411 cases had missing data on at least one study variable and were excluded using complete case analysis (listwise deletion), yielding a final analytic sample of 13,571. Complete case analysis was used for transparency and reproducibility. However, it can reduce precision and may introduce bias if missingness is systematically related to the outcome, predictors included in the model, or unmeasured factors associated with these variables [46].

### 2.2. Measures

#### 2.2.1. Outcome Variable: Child Flourishing and Family Resilience Status

The outcome variable for this study was created using two resilience measures: child flourishing and family resilience. First, child flourishing was measured using three items: (1) showed interest and curiosity in learning new things, (2) worked to finish the tasks they started, and (3) stayed calm and in control when faced with a challenge. The items were rated on a 4-point Likert scale ranging from “always” (=1) to “never” (=4). Cronbach’s alpha for the child flourishing measure in this sample was 0.72. Following the NSCH’s predefined scoring, child flourishing was coded as a dichotomous indicator, with children classified as flourishing if their parents responded “always” or “usually” to all three items.

Family resilience was measured using four items: (1) talked together about what to do when the family faces problems, (2) worked together to solve the problem when your family faces problems, (3) knew we had strengths to draw on when the family faces problems, and (4) stayed hopeful even in difficult times when the family faced problems. Items were rated on a 4-point Likert scale ranging from “all of the time” (=1) to “none of the time” (=4). Cronbach’s alpha for the family resilience measure in this sample was 0.90. Using the NSCH predefined scoring, the family resilience measure was coded as a dichotomous indicator, with families classified as resilient if parents responded, “all of the time” or “most of the time” to all four items.

Finally, these two dichotomous resilience measures were combined to create a categorical outcome variable with four mutually exclusive groups: Neither Child Flourishing nor Family Resilience (reference group), Child Flourishing Only, Family Resilience Only, and Child Flourishing and Family Resilience.

#### 2.2.2. Adverse Childhood Experiences

The 2024 NSCH included ten lifetime reports of ACEs: (1) economic hardship, (2) parent divorce or separation, (3) parent death, (4) parent time in jail, (5) domestic violence, (6) mental illness, (7) substance misuse, (8) neighborhood violence, (9) racial discrimination, and (10) discrimination due to a health condition or disability. Consistent with prior research emphasizing the dose response pattern [1,22,23], a cumulative ACEs measure was created and classified into four categories using 1 ACE exposure as the reference group: 1, 2, 3, and 4 or more ACE exposures.

#### 2.2.3. Individual-Level Predictors

Child sex was measured using the parent report (0 = “female”, 1 = “male”). Race/ethnicity was dummy coded into four categories, using “white/non-Hispanic” as the reference group: “white/non-Hispanic”, “black/non-Hispanic”, “Hispanic”, and “other”. Child age was treated as a continuous variable, measured in years, with a range from 6 to 17 years. Access to health care was measured using the NSCH medical home composite, derived from 14 survey items across five components: (1) having a personal doctor or nurse, (2) having a usual source of care when sick, (3) receiving family-centered care, (4) experiencing no issues obtaining necessary referrals, and (5) receiving effective care coordination when needed. Consistent with NSCH guidance, children who met the criteria for at least one of these components were classified as having access to health care. All others were classified as not having access to health care.

#### 2.2.4. Parent and Family Predictors

Parent educational attainment was a binary variable indicating whether the parent had a “high school degree or below” (=1) versus “some college or higher” (=0). Family structure was classified into four categories, using “two parents/married” as the reference group: “two parents/married”, “two parents/not married”, “single parent”, and “other family type”. Household income was estimated using the imputed federal poverty level (FPL) and then classified into four groups: “0–99 FPL”, “100–199% FPL”, “200–399% FPL”, “400% FPL or higher” (reference group). Lastly, parenting stress was measured with three items capturing perceived stress during the past month: (1) the child was much harder to care for than most children during the past month, (2) the child did things that bothered them during the past month, and (3) was angry with the child during the past month. Responses ranged from “never” (=1) to “usually or always” (=4). Cronbach’s alpha for the parenting stress measure in this sample was 0.81. Consistent with the NSCH predefined scoring, parenting stress was coded as a dichotomous indicator, with parents classified as experiencing parenting stress if they responded “always” or “usually” to all three items.

#### 2.2.5. School and Community Predictors

School safety. School safety was assessed with a single item: “This child is safe at school.” Response options ranged from 1 = “definitely agree” to 4 = “definitely disagree.” For analysis, responses of “definitely agree” or “somewhat agree” were coded as indicating school safety.

Neighborhood safety. Neighborhood safety was assessed with a single item: “This child is safe in our neighborhood.” Response options ranged from 1 = “definitely agree” to 4 = “definitely disagree.” As with school safety, responses of “definitely agree” and “somewhat agree” were coded as indicating neighborhood safety.

Supportive neighborhood. Supportive neighborhood was measured using three items: (1) people in this neighborhood help each other out, (2) we watch out for each other’s children in this neighborhood, and (3) when we encounter difficulties, we know where to go for help in our community. Responses were recorded on a four-point scale ranging from “definitely agree” (=1) to “definitely disagree” (=4). Cronbach’s alpha for the supportive neighborhood measure in this sample was 0.78. Following the NSCH’s predefined scoring, children were classified as living in a supportive neighborhood if parents responded “definitely agree” to at least one item and “somewhat agree” or “definitely agree” to the other two items.

Neighborhood amenities. Neighborhood amenities were measured as a composite indicator derived from the presence of four neighborhood amenities: (1) sidewalks or walking paths, (2) parks or playgrounds, (3) recreation centers, community centers, or boys’ and girls’ clubs, and (4) libraries or bookmobiles. Each item was coded as “1” if present and “0” otherwise. A binary variable was then constructed to indicate the presence of neighborhood amenities (1 = all four amenities present, 0 = otherwise)

Distracting neighborhood conditions. Distracting neighborhood conditions were measured as a composite indicator based on three detracting characteristics present in the neighborhood: (1) litter or garbage on the street or sidewalk, (2) poorly maintained or rundown housing, and (3) vandalism, such as broken windows or graffiti. Each item was coded as “1” if present and “0” otherwise. A binary variable was then constructed to indicate distracting neighborhood conditions (1 = all three detracting factors present; 0 = otherwise).

### 2.3. Analysis

Frequency distributions were examined for study variables. A multinomial regression model was performed to identify socio-ecological predictors of child flourishing and family resilience status among children who had at least one ACE exposure. In addition to reporting adjusted relative risk ratios (RRRs), average marginal effects (AMEs) were estimated to quantify the average change in the predicted probability of each outcome category associated with a one-unit increase in continuous predictors or a change from the reference category for categorical predictors. Data management was conducted in SPSS (version 28) and primary analyses were conducted in STATA (version 19). Cluster and sampling weights were used to account for the complex sampling design of the survey. Results were considered significant at *p* < 0.05. Because the current study is a secondary analysis of publicly available data, the institutional review board of the Name Redacted University deemed this study exempt from human subject review.

## 3. Results

### 3.1. Descriptive Statistics of Study Sample by Child Flourishing and Family Resilience Status

Table 1 presents descriptive characteristics for the full sample and by the four child flourishing and family resilience group categories. In the full sample, 46.5% (n = 5845) were classified in the child flourishing and family resilience group; 33.3% (n = 4896) were in the family resilience only group; 13.6% (n = 2002) were in the neither child flourishing nor family resilience group; and 6.6% (n = 828) were in the child flourishing only group. Slightly more than half of the sample (52.2%) were male. Nearly half of the sample were White/Non-Hispanic (44.4%), followed by Hispanic (27.8%), Black/Non-Hispanic (15.8%), and Other (12.0%). Children’s age ranged from 6 to 17, with a mean age of 12 years. Over one-third of children had access to health care at the time of the survey (37.0%). Approximately one-third of parents had a high school degree or below (31.9%). About half of the sample lived with two parents (married or not married; 50.7%) and 45.3% reported the household income was less than 199% of the federal poverty level (FPL). Overall, about 8% of parents reported parenting stress. For the school and community characteristics, 94.3% reported their child was safe at school and 93.3% reported their child was safe in their neighborhood. Slightly less than half of the children lived in a neighborhood that parents perceived as not supportive (47.2%). About one-third of the children lived in neighborhoods with all four amenities (34.5%) and nearly one-third lived in neighborhoods with all three distracting conditions (29.6%).

Descriptive statistics by child and family resilience group categories are comparable across groups with a few exceptions. For example, access to health care was lowest among children in the neither child flourishing nor family resilience group (27.3%). Parenting stress was highest among parents of children in the neither child flourishing nor family resilience group (23.0%) and lowest among parents of children in the child flourishing and family resilience group (1.4%). Compared to children in the child flourishing and family resilience group, children in the neither child flourishing nor family resilience group were less likely to be reported as safe at school (88.4% vs. 97.1%) and in their neighborhoods (88.6% vs. 94.9%). They also differed in neighborhood context, with a higher proportion living in neighborhoods with all three distracting conditions (38.8% vs. 27.0%) and in neighborhoods perceived by parents as not supportive (56.8% vs. 27.3%).

### 3.2. Prevalence of Individual and Cumulative ACEs

Table 2 reports the prevalence of individual and cumulative ACEs experienced by children in the sample. Overall, the most prevalent type of ACE was parent or guardian divorce or separation (58.5%), whereas the least prevalent was being treated or judged unfairly because of the child’s health condition or disability (8.6%). Children in the neither child flourishing nor family resilience group reported the highest prevalence rates across many individual ACEs, with a few exceptions. Specifically, the child flourishing only group also reported the highest prevalence rates of economic hardship (40.8%) and parent or guardian divorce or separation (59.5%) and the family resilience only group reported the highest prevalence rates of parental incarceration (17.2%). Regarding cumulative ACE exposure, 52.2% of the children had one ACE, 21.9% had 2 ACEs, and 26.0% had 3 or more ACEs. Children in the neither child flourishing nor family resilience group had the highest cumulative exposure, with 23.8% reporting four or more ACEs, followed by the family resilience only group (17.9%), the child flourishing only group (12.5%), and the child flourishing and family resilience group (9.5%).

### 3.3. Associations of Socio-Ecological Factors with Child Flourishing and Family Resilience Status

Table 3 presents results from the multinomial logistic regression examining socio-ecological factors associated with child flourishing and family resilience status, using the neither child flourishing nor family resilience group as the reference. Prior to estimating the multinomial model, sensitivity analyses were conducted using continuous measures of child flourishing and family resilience to assess whether findings were robust to alternative outcome specifications. Results from these continuous outcome models were substantively consistent with the primary categorical analysis, supporting the decision to operationalize child flourishing and family resilience as dichotomous indicators and to model their combined status using four profile categories. The sensitivity analysis results are presented in Supplemental Table S1.

#### 3.3.1. Individual-Level Predictors

At the individual level, several predictors were significantly associated with resilience status. Male sex was associated with lower odds of being in the child flourishing and family resilience group (RRR = 0.79, 95% CI = 0.64–0.98). Race and ethnicity were also associated with membership in the child flourishing and family resilience group, such that Black non-Hispanic (RRR = 1.41, 95% CI = 1.03–1.92) and Hispanic children (RRR = 1.35, 95% CI = 1.02–1.79) had higher odds of being in this group compared with White non-Hispanic children. Additionally, children in the other race/ethnicity category had higher odds of being in the child flourishing only group (RRR = 1.71, 95% CI = 1.08–2.70). Finally, older age was associated with lower odds of being in the family resilience only group (RRR = 0.96, 95% CI = 0.93–0.99). Access to health care was associated with higher odds of being in the child flourishing and family resilience group (RRR = 1.53, 95% CI = 1.22–1.91), relative to the reference group.

#### 3.3.2. Parent and Family-Level Predictors

At the parent and family level, parenting stress was a significant predictor of resilience status. Higher parenting stress was associated with lower odds of being in the child flourishing only group (RRR = 0.23, 95% CI = 0.10–0.52), the family resilience only group (RRR = 0.56, 95% CI = 0.45–0.71), and the child flourishing and family resilience group (RRR = 0.06, 95% CI = 0.04–0.09), relative to the reference group. Family structure was also associated with membership in the child flourishing and family resilience group. Compared with children living with two married parents, children living in single-parent households (RRR = 1.28, 95% CI = 1.02–1.60) and those in other family types (RRR = 1.90, 95% CI = 1.25–2.88) had higher odds of being in the child flourishing and family resilience group. Parental educational attainment and household income were not significantly associated with resilience status.

#### 3.3.3. School and Community-Level Predictors

Several school and neighborhood factors were significantly associated with resilience status. School safety was associated with higher odds of being in the child flourishing and family resilience group (RRR = 1.85, 95% CI = 1.21–2.82), relative to the reference group. Living in a supportive neighborhood was associated with higher odds of being in the child flourishing only group (RRR = 1.63, 95% CI = 1.14–2.31), the family resilience only group (RRR = 1.74, 95% CI = 1.41–2.15), and the child flourishing and family resilience group (RRR = 3.03, 95% CI = 2.43–3.77). Neighborhood amenities were also associated with higher odds of being in the child flourishing and family resilience group (RRR = 1.27, 95% CI = 1.03–1.57). In contrast, distracting neighborhood conditions were associated with lower odds of being in the family resilience only group (RRR = 0.78, 95% CI = 0.62–0.98).

#### 3.3.4. Cumulative ACEs and Child Flourishing and Family Resilience Status

Cumulative ACE exposure demonstrated a dose–response association with resilience status. Compared with children with one ACE, children with two, three, and four or more ACEs had lower odds of being in the child flourishing and family resilience group (RRR = 0.64, 95% CI = 0.50–0.82; RRR = 0.40, 95% CI = 0.26–0.62; and RRR = 0.30, 95% CI = 0.23–0.41, respectively). Children with four or more ACEs also had lower odds of being in the child flourishing only group (RRR = 0.42, 95% CI = 0.26–0.68) and lower odds of being in the family resilience only group (RRR = 0.74, 95% CI = 0.57–0.98), relative to the reference group.

#### 3.3.5. Predicted Probabilities of Child Flourishing and Family Resilience Status

Table 4 reports the results of average marginal effects (AMEs) from the multinomial logistic regression model. Since this study focuses on socioecological factors predicting the child flourishing and family resilience status, the table only summarizes AMEs for key socioecological predictors. The results of the full model are provided in Supplemental Table S2.

Among children with at least one ACE, AMEs from the multinomial model indicated that parenting stress was associated with significant shifts in outcome probabilities. Specifically, holding other variables constant, parenting stress was associated with an 18 percentage point higher predicted probability of being in the neither child flourishing nor family resilience group (AME = 0.18, SE = 0.01, *p* < 0.001) and a 30 percentage point higher predicted probability of being in the family resilience only group (AME = 0.30, SE = 0.03, *p* < 0.001), as well as a 49 percentage point lower predicted probability of being in the child flourishing and family resilience group (AME = −0.49, SE = 0.05, *p* < 0.001). The association with the child flourishing only group was not statistically significant. In contrast, holding other variables constant, higher school safety was associated with a 4 percentage point lower predicted probability of being in the neither child flourishing nor family resilience group (AME = −0.04, SE = 0.02, *p* < 0.05) and an 11 percentage point higher predicted probability of being in the child flourishing and family resilience group (AME = 0.11, SE = 0.04, *p* < 0.01), with no statistically significant differences for the child flourishing only group or the family resilience only group.

Similarly, holding other variables constant, living in a supportive neighborhood was associated with a 9 percentage point lower predicted probability of being in the neither child flourishing nor family resilience group (AME = −0.09, SE = 0.01, *p* < 0.001) and a 5 percentage point lower predicted probability of being in the family resilience only group (AME = −0.05, SE = 0.01, *p* < 0.01), and a 15 percentage-point higher predicted probability of being in the child flourishing and family resilience group (AME = 0.15, SE = 0.02, *p* < 0.001). Neighborhood amenities showed a small but statistically significant association with a 3 percentage point higher predicted probability of being in the child flourishing and family resilience group (AME = 0.03, SE = 0.02, *p* < 0.05), whereas distracting neighborhood conditions and neighborhood safety were not significantly associated with predicted probabilities of any outcome group.

Finally, holding other variables constant, compared with children in the one ACE group, higher adversity burden demonstrated a dose–response pattern in predicted probabilities. Specifically, children with two ACEs had a 3 percentage point higher predicted probability of being in the neither child flourishing nor family resilience group (AME = 0.03, SE = 0.01, *p* < 0.05) and a 5 percentage point higher predicted probability of being in the family resilience only group (AME = 0.05, SE = 0.02, *p* < 0.05), and an 8 percentage point lower predicted probability of being in the child flourishing and family resilience group (AME = −0.08, SE = 0.02, *p* < 0.001). Children with three ACEs had a 6 percentage point higher predicted probability of being in the neither child flourishing nor family resilience group (AME = 0.06, SE = 0.02, *p* < 0.05) and an 8 percentage point higher predicted probability of being in the family resilience only group (AME = 0.08, SE = 0.03, *p* < 0.01), and a 15 percentage point lower predicted probability of being in the child flourishing and family resilience group (AME = −0.15, SE = 0.03, *p* < 0.001). Finally, children with four or more ACEs had a 9 percentage point higher predicted probability of being in the neither child flourishing nor family resilience group (AME = 0.09, SE = 0.02, *p* < 0.001) and a 12 percentage point higher predicted probability of being in the family resilience only group (AME = 0.12, SE = 0.02, *p* < 0.001), and a 19 percentage point lower predicted probability of being in the child flourishing and family resilience group (AME = −0.19, SE = 0.02, *p* < 0.001). No significant differences were observed in the predicted probability of being in the child flourishing only group across ACE categories.

## 4. Discussion

This study examined socioecological predictors of child flourishing and family resilience status among U.S. school-aged children with adverse childhood experiences (ACEs), using nationally representative 2024 NSCH data. The findings indicate that nearly half of the children with ACEs in the sample exhibited both child flourishing and family resilience, suggesting that many families possess protective factors that help them thrive in challenging circumstances. At the same time, the presence of discordant resilience groups indicates that child flourishing and family resilience do not necessarily co-occur. In particular, one third of children were classified as living in resilient families without themselves flourishing (33.3%), and a smaller subgroup was classified as flourishing in the context of limited family resilience (6.6%). Most importantly, about 14% of children fell into the most vulnerable group, demonstrating neither child flourishing nor family resilience. This subgroup warrants particular attention, as they may be at heightened risk for poor long-term outcomes. This heterogeneity across resilience status groups underscores the conceptual importance of treating child-level and family-level adaptation as related but distinct domains, with protective processes potentially operating in different parts of the social ecology [26,29,30,31].

The findings also highlight that socioecological conditions beyond the household are closely associated with resilience status group membership. Perceived school safety, supportive neighborhood conditions, and neighborhood amenities were consistently associated with a greater likelihood of membership in the group characterized by both child flourishing and family resilience. These findings are consistent with ecological models of resilience emphasizing that safe, supportive environments and reliable access to social resources can buffer stress exposure and support positive adaptation, especially during the school-aged developmental period when children spend substantial time in school and increasingly engage with peers and community settings [19,28,38,39,47]. School safety may represent more than physical protection, capturing aspects of school climate such as predictability, connectedness, and adult monitoring, which can promote engagement and support self-regulation in the context of stress. Similarly, supportive neighborhood processes likely reflect social cohesion and informal support, including mutual aid and collective efficacy, which can reduce caregiver isolation and strengthen access to practical and emotional resources. Neighborhood amenities may further support resilience by providing spaces and opportunities for recreation, enrichment, and prosocial interaction (e.g., parks, libraries, community centers), thereby facilitating connection to community-based resources and supportive relationships that promote thriving.

Notably, neighborhood safety and distracting neighborhood conditions showed weaker and less consistent relationships with resilience status group membership after accounting for other covariates. One interpretation is that relational and resource-oriented dimensions of neighborhood context may be more proximal to the resilience processes captured by the NSCH measures than broader perceptions of safety or physical disorder. Another possibility concerns measurement sensitivity. A single-item measure of neighborhood safety may have limited variability or may overlap conceptually with school safety, while indicators of distracting neighborhood conditions may not fully capture chronic stress exposure in ways most relevant to child flourishing and family resilience.

Although the present study emphasizes socioecological factors, cumulative childhood adversity and caregiver strain provide important context for interpreting the ecological findings. First, the dose–response pattern for cumulative ACE exposure remained evident, with higher ACE burden associated with a lower likelihood of membership in groups characterized by child flourishing and/or family resilience and a higher likelihood of membership in the neither child flourishing nor family resilience group. This is consistent with cumulative risk perspectives, suggesting that as adversity accumulates, it may exceed the buffering capacity of available protective resources. In addition, parenting stress was strongly related to resilience status group membership, aligning with stress process frameworks in which adversity increases caregiver strain and can undermine parenting resources that support child regulation and engagement [36,37]. These family stress processes may interact with socioecological conditions. Supportive schools and neighborhoods may partially compensate for caregiver strain by providing consistent routines, supportive adults, and access to social resources, but they may not fully offset the effects of high cumulative adversity or sustained parenting stress. This integrative framing may help explain why context-focused supports are necessary but may be insufficient without concurrent efforts to reduce caregiver strain and strengthen family resources.

The study’s findings have several important implications for practice and policy aimed at supporting children and families exposed to adverse childhood experiences (ACEs). First, the discordant resilience groups suggest that services should be aligned with the domain in which adaptation appears constrained. For example, children who flourish despite limited family resilience may be drawing on protective resources outside the home, such as schools, peers, mentors, or healthcare, whereas resilient family coping processes may not fully buffer child-level challenges such as learning difficulties, behavioral health needs, or stressors occurring in other settings. Accordingly, a coordinated, multi-level strategy is needed to strengthen protective factors across the social ecology and to match supports to identified needs [19,47]. At the individual and family levels, family-centered interventions that reduce parenting stress and promote relational health, including strengthening parent–child relationships, enhancing caregivers’ coping and emotion-regulation skills, and expanding social support networks, may be especially important for children who are flourishing but whose caregivers have limited coping resources [30,31]. In contrast, when families demonstrate resilient coping, but children are not flourishing, child-focused supports may be especially critical, including social-emotional learning, academic support, and access to behavioral health services that directly address self-regulation, emotional distress, or school functioning [48,49].

More broadly, schools play a critical role in resilience promotion. Policies and programs that strengthen school climate and safety such as bullying prevention, predictable routines, positive behavior supports, and initiatives that enhance adult-student connectedness and monitoring may be particularly important for children with ACE exposure during the school-aged period, when schools serve as a central developmental context [49]. At the community level, efforts that build social cohesion, collective efficacy, and informal helping networks may reduce caregiver isolation and expand access to practical and emotional supports, thereby reinforcing both child and family adaptation. In addition, investments that increase access to neighborhood amenities (e.g., parks, recreation spaces, libraries, and other community resources) may facilitate connection, engagement, and supportive relationships that support thriving. Together, these school- and community-level investments can help create safe, stable environments that support resilience, expand positive developmental opportunities, and mitigate the harmful effects of neighborhood stressors [38,39,47].

It is important to acknowledge the limitations of this study. First, the NSCH data are cross-sectional, which limits the ability to establish causal relationships between socioecological factors and resilience. Second, the data rely on self-reports from primary caregivers, which may introduce recall bias, social desirability bias, and other forms of measurement error. For example, caregivers may underreport children’s ACE exposure or overreport positive functioning and family coping. Such misclassification could lead to underestimation of ACE prevalence and potentially attenuate observed associations between socioecological factors and resilience status. Third, although the analyses adjusted for a range of relevant covariates, the study did not account for all potential confounding factors, and some associations may reflect unmeasured influences. Fourth, the operationalization of child flourishing and family resilience, while consistent with prior NSCH-based research, used relatively stringent dichotomization thresholds. These conservative criteria may capture more stable or pronounced forms of flourishing and resilience and could yield lower prevalence estimates than would be observed with more inclusive cut points. Importantly, stringent thresholds may also influence classification into the four resilience profiles. For example, some children or families who show moderate strengths may be categorized as not flourishing or not resilient, potentially shifting individuals from the child flourishing only or family resilience only groups into the neither group and reducing the size of the both group. Relatedly, the study relied on a limited set of indicators for both constructs, which may not fully represent other important dimensions of child flourishing and family resilience. Finally, resilience and flourishing are inherently dynamic processes that unfold over time [26]. These measures reflect a snapshot at one time point rather than developmental trajectories, and resilience profiles may shift as children mature and as family, school, and community contexts change.

Despite these limitations, the study makes a valuable contribution by addressing both child and family resilience, offering a more holistic understanding of resilience in the context of ACEs. This research provides a strong foundation for developing targeted interventions and policies aimed at promoting well-being among children and families facing adversity. Future research should consider longitudinal designs to better explore how resilience develops over time and how different factors interact to influence long-term outcomes. Additionally, qualitative research could offer deeper insights into the lived experiences of children and families, providing a more nuanced and comprehensive understanding of resilience.

## 5. Conclusions

Building on prior research that recognizes resilience as a critical factor mitigating the effects of ACEs, the current study delves into socioecological predictors of child flourishing and family resilience among U.S. school-aged children with ACE exposure. The findings underscore that resilience is multidimensional, with child flourishing and family resilience not always co-occurring, and that modifiable contexts such as school safety and supportive neighborhood conditions are closely linked to resilience status group membership. These results suggest the importance of multilevel strategies that strengthen children’s everyday environments while also supporting family resources, particularly for those in the most vulnerable status. Future research should use longitudinal designs to examine transitions across resilience groups over time and incorporate qualitative approaches to clarify the mechanisms through which family, school, and community contexts shape resilience processes.

## Figures and Tables

**Table 1:** Descriptive Statistics by Child Flourishing and Family Resilience Status.

	Full Sample (N = 13,571)	Neither Child Flourishing nor Family Resilience (n = 2002)	Child Flourishing Only (n = 828)	Family Resilience Only (n = 4896)	Child Flourishing and Family Resilience (n = 5845)
Individual-level Predictors					
Child sex (%, male)	52.2	53.9	50.4	57.2	48.3
Race/Ethnicity					
White, Non-Hispanic (%)	44.4	42.7	36.3	50.4	41.8
Black, Non-Hispanic (%)	15.8	15.4	17.2	13.8	17.0
Hispanic (%)	27.8	29.4	29.3	23.6	30.1
Other race/ethnicity (%)	12.0	12.6	17.2	12.1	11.1
Age, mean (SD)	12.1 (3.4)	12.4 (3.3)	12.7 (3.3)	11.9 (3.4)	12.1 (3.3)
Access to health care (%)	37.0	27.3	32.8	35.2	41.7
Parent and Family Predictors					
Parental educational attainment (%, high school degree or below)	31.9	35.6	36.9	27.6	33.2
Family Structure					
Two parents, married (%)	42.2	40.6	38.2	47.5	39.6
Two parents, not married (%)	8.5	10.5	6.1	8.1	8.6
Single parent (%)	42.2	43.0	50.0	36.9	44.6
Other family types (%)	7.1	5.9	5.6	7.6	7.3
Household Income					
0–99% FPL (%)	19.9	22.1	23.3	19.9	18.7
100–199% FPL (%)	25.4	27.0	31.9	23.1	25.6
200–399% FPL (%)	29.0	29.3	22.8	29.4	29.6
400% FPL or higher (%)	25.7	21.6	22.1	27.6	26.1
Parenting stress (%)	8.8	23.0	5.9	13.9	1.4
School and Community Predictors					
School safety (%)	94.3	88.4	91.2	93.4	97.1
Neighborhood safety (%)	93.3	88.6	90.0	93.7	94.9
Supportive neighborhood (%)	47.2	27.3	37.4	43.8	56.8
Neighborhood amenities (%)	34.5	29.9	31.1	33.9	36.8
Distracting neighborhood conditions (%)	29.6	38.8	36.5	28.2	27.0
N (% of Full Sample)	13,571	2002 (13.6)	828 (6.6)	4896 (33.3)	5845 (46.5)

SD = Standard Deviation. FPL = Federal Poverty Level. Weighted proportions are presented. Other race/ethnicity category includes American Indian or Alaska Native, Asian, Native Hawaiian and Pacific Islander, multiracial. Other family types include grandparents, foster care parents, and relatives. Given the significant number of missing values of household income, 6 imputed FPL variables provided by the NSCH were incorporated in the analyses using multiple imputation function.

**Table 2:** Prevalence of Individual and Cumulative Adverse Childhood Experiences by Child Flourishing and Family Resilience Status.

	Full Sample (N = 13,571)	Neither Child Flourishing nor Family Resilience (n = 2002)	Child Flourishing Only (n = 828)	Family Resilience Only (n = 4896)	Child Flourishing and Family Resilience (n = 5845)
Individual ACEs					
Hard to cover basics like food or housing (%)	33.8	40.5	40.8	33.3	31.2
Parent or guardian divorced or separated (%)	58.5	59.0	59.5	57.8	58.8
Parent or guardian died (%)	9.2	11.1	7.2	8.9	9.1
Parent or guardian served time in jail (%)	15.1	17.1	11.0	17.2	13.7
Saw or heard parents or adults slap, hit, kick, or punch one another in the home (%)	11.5	16.8	15.4	14.3	7.4
Lived with anyone who was mentally ill, suicidal, or severely depressed (%)	21.6	31.4	22.4	25.1	16.2
Lived with anyone who had a problem with alcohol or drugs (%)	21.4	28.7	20.8	25.5	16.5
Was a victim of violence or witnessed violence in the neighborhood (%)	11.4	15.8	13.5	13.2	8.5
Treated or judged unfairly because of his or her race or ethnic group (%)	12.8	15.9	10.6	13.8	11.6
Treated or judged unfairly because of his or her health condition or disability (%)	8.6	15.8	3.1	14.0	3.3
Cumulative ACEs					
1 (%)	52.2	38.7	47.8	46.7	60.6
2 (%)	21.9	21.6	25.2	22.7	20.9
3 (%)	11.6	16.0	14.5	12.7	9.0
4 or more (%)	14.4	23.8	12.5	17.9	9.5

Weighted proportions are presented.

**Table 3:** Socioecological Predictors of Child Flourishing and Family Resilience Status among Children with a History of ACEs (N = 13,571).

	Child Flourishing Only	Family Resilience Only	Child Flourishing and Family Resilience
	RRR (95% CI)	RRR (95% CI)	RRR (95% CI)
Individual Predictors			
Sex			
Female	1.00 (Reference)	1.00 (Reference)	1.00 (Reference)
Male	0.85 (0.61, 1.18)	1.14 (0.92, 1.40)	0.79 * (0.64, 0.98)
Race/Ethnicity			
White, Non-Hispanic	1.00 (Reference)	1.00 (Reference)	1.00 (Reference)
Black, Non-Hispanic	1.32 (0.84, 2.08)	0.91 (0.67, 1.23)	1.41 * (1.03, 1.92)
Hispanic	1.18 (0.77, 1.81)	0.84 (0.64, 1.11)	1.35 * (1.02, 1.79)
Other race/ethnicity	1.71 * (1.08, 2.70)	0.86 (0.64, 1.15)	1.02 (0.76, 1.36)
Age (range = 6–17 y)	1.03 (0.98, 1.09)	0.96 ** (0.93, 0.99)	0.98 (0.95, 1.01)
Access to health care	1.19 (0.86, 1.65)	1.19 (0.96, 1.48)	1.53 *** (1.22, 1.91)
Parent and Family Predictors			
Parental Educational Attainment			
High school degree or below	0.96 (0.64, 1.44)	0.79 (0.61, 1.01)	1.01 (0.78, 1.31)
Some college or higher	1.00 (Reference)	1.00 (Reference)	1.00 (Reference)
Family Structure			
Two parents, married	1.00 (Reference)	1.00 (Reference)	1.00 (Reference)
Two parents, not married	0.59 (0.31, 1.12)	0.70 (0.44, 1.10)	0.86 (0.54, 1.38)
Single parent	1.29 (0.92, 1.81)	0.85 (0.68, 1.06)	1.28 * (1.02, 1.60)
Other family types	1.24 (0.62, 2.48)	1.29 (0.87, 1.91)	1.90 ** (1.25, 2.88)
Household Income			
0–99% FPL	1.29 (0.68, 2.45)	1.12 (0.79, 1.60)	1.03 (0.73, 1.43)
100–199% FPL	1.40 (0.86, 2.29)	0.98 (0.73, 1.32)	1.12 (0.81, 1.54)
200–399% FPL	0.86 (0.54, 1.37)	0.92 (0.71, 1.19)	1.02 (0.79, 1.31)
400% FPL or higher	1.00 (Reference)	1.00 (Reference)	1.00 (Reference)
Parenting stress	0.23 *** (0.10, 0.52)	0.56 *** (0.45, 0.71)	0.06 *** (0.04, 0.09)
School and Community Predictors			
School safety	0.98 (0.45, 2.14)	1.15 (0.83, 1.58)	1.85 ** (1.21, 2.82)
Neighborhood safety	0.92 (0.46, 1.83)	1.13 (0.78, 1.62)	0.99 (0.65, 1.49)
Supportive neighborhood	1.63 ** (1.14, 2.31)	1.74 *** (1.41, 2.15)	3.03 *** (2.43, 3.77)
Neighborhood amenities	1.05 (0.76, 1.44)	1.15 (0.94, 1.42)	1.27 * (1.03, 1.57)
Distracting neighborhood conditions	1.03 (0.72, 1.49)	0.78 * (0.62, 0.98)	0.88 (0.70, 1.12)
Cumulative ACEs			
1 ACE	1.00 (Reference)	1.00 (Reference)	1.00 (Reference)
2 ACEs	0.91 (0.62, 1.33)	0.91 (0.71, 1.17)	0.64 *** (0.50, 0.82)
3 ACEs	0.69 (0.38, 1.26)	0.76 (0.52, 1.11)	0.40 *** (0.26, 0.62)
4 or more ACEs	0.42 *** (0.26, 0.68)	0.74 * (0.57, 0.98)	0.30 *** (0.23, 0.41)

RRR = Relative Risk Ratio; CI = Confidence Interval; FPL = Federal Poverty Level; ACE = Adverse Childhood Experiences. Reference = Neither Child Flourishing nor Family Resilience Group. * *p* < 0.05; ** *p* < 0.01; *** *p* < 0.001.

**Table 4:** Predicted Probabilities of Child Flourishing and Family Resilience Status from Multinomial Logistic Regression Model (N = 13,571).

	Neither Child Flourishing nor Family Resilience		Child Flourishing Only		Family Resilience Only		Child Flourishing and Family Resilience	
	AME	SE	AME	SE	AME	SE	AME	SE
Parenting stress	0.18 ***	0.01	0.01	0.02	0.30 ***	0.03	−0.49 ***	0.05
School safety	−0.04 *	0.02	−0.02	0.02	−0.06	0.03	0.11 **	0.04
Neighborhood safety	−0.00	0.02	−0.01	0.02	0.03	0.03	−0.02	0.04
Supportive neighborhood	−0.09 ***	0.01	−0.02	0.01	−0.05 **	0.01	0.15 ***	0.02
Neighborhood amenities	−0.02	0.01	−0.01	0.01	−0.00	0.01	0.03 *	0.02
Distracting neighborhood conditions	0.02	0.01	0.01	0.01	−0.04	0.02	0.01	0.02
Cumulative ACEs								
2 ACEs	0.03 *	0.01	0.01	0.01	0.05 *	0.02	−0.08 ***	0.02
3 ACEs	0.06 *	0.02	0.01	0.02	0.08 **	0.03	−0.15 ***	0.03
4 or more ACEs	0.09 ***	0.02	−0.01	0.01	0.12 ***	0.02	−0.19 ***	0.02

AME = Average Marginal Effect; SE = Standard Error. * *p* < 0.05; ** *p* < 0.01; *** *p* < 0.001.

## Data Availability

Data used in the study are available upon request to the corresponding author.

## Supplementary Materials

The following supporting information can be downloaded at: https://www.mdpi.com/article/10.3390/ijerph23030277/s1, Table S1: OLS Regression Models Predicting Child Flourishing and Family Resilience; Table S2: Predicted Probabilities of Child Flourishing and Family Resilience Status from Multinomial Logistic Regression-Full Model (N = 13,571).
