# Supplementary material for: Socioecological Predictors of Child Flourishing and Family Resilience Status Among Children with Adverse Childhood Experiences
Source: Int J Environ Res Public Health. 2026 Feb 24;23(3):277. doi: 10.3390/ijerph23030277 (PMC13026905; doi:10.3390/ijerph23030277)
Supplement: Supplementary file 1 [file ijerph-23-00277-s001.zip › Supplemental Table 1.docx]

Supplemental Table 1. OLS Regression Models Predicting Child Flourishing and Family Resilience

|  | Child Flourishing  (N = 13,571) | | Family Resilience (N = 13,571) | |
| --- | --- | --- | --- | --- |
|  | b | SE | b | SE |
| **Individual Predictors** |  |  |  |  |
| Sex (Male = 1) | -0.35*** | (0.06) | 0.07 | (0.08) |
| Race/Ethnicity (reference group = White, Non-Hispanic) |  |  |  |  |
| Hispanic | 0.42*** | (0.08) | 0.21* | (0.10) |
| Black, Non-Hispanic | 0.52*** | (0.10) | 0.32** | (0.12) |
| Other race/ethnicity | 0.22** | (0.08) | -0.12 | (0.12) |
| Child age (years) | -0.03* | (0.01) | -0.05*** | (0.01) |
| Access to health care (Yes = 1) | 0.17** | (0.06) | 0.18* | (0.08) |
| **Parent and Family Predictors** |  |  |  |  |
| Parent education (high school or less, reference group = Some college or higher) | 0.24** | (0.08) | 0.10 | (0.10) |
| Family Structure (reference group = Two parents, married) |  |  |  |  |
| Two parents, not married | 0.06 | (0.13) | -0.04 | (0.16) |
| Single parent | 0.28*** | (0.07) | 0.07 | (0.09) |
| Other family types | 0.14 | (0.14) | 0.25 | (0.14) |
| Household Income (reference group = 400% FPL or higher) |  |  |  |  |
| 0-99% FPL | -0.04 | (0.12) | 0.15 | (0.15) |
| 100-199% FPL | 0.07 | (0.09) | 0.09 | (0.11) |
| 200-399% FPL | 0.05 | (0.07) | 0.02 | (0.09) |
| Parenting stress (Yes = 1) | -2.01*** | (0.11) | -0.76*** | (0.14) |
| **School and Community Predictors** |  |  |  |  |
| School safety (Yes = 1) | 0.52*** | (0.13) | 0.59** | (0.22) |
| Neighborhood safety (Yes = 1) | -0.10 | (0.13) | 0.01 | (0.21) |
| Supportive neighborhood (Yes = 1) | 0.62*** | (0.06) | 1.00*** | (0.08) |
| Neighborhood amenities (Yes = 1) | 0.06 | (0.06) | 0.10 | (0.07) |
| Distracting neighborhood conditions (Yes = 1) | 0.03 | (0.08) | -0.15 | (0.10) |
| **Cumulative ACEs (reference group = 1 ACE)** |  |  |  |  |
| 2 ACEs | -0.24** | (0.07) | -0.34*** | (0.09) |
| 3 ACEs | -0.46*** | (0.13) | -0.50** | (0.18) |
| 4 or more ACEs | -0.67*** | (0.09) | -0.51*** | (0.12) |
| Constant | 8.67*** | (0.23) | 9.09*** | (0.35) |
| Model *F* Statistic | 45.81 | | 18.67 | |
| Model df (numerator) | 22 | | 22 | |
| Model df (denominator) | 12513.7 | | 12754.1 | |
| p value | > 0.001 | | > 0.001 | |

SE = Standard Error. * p < .05; ** p < .01; *** p < .001.
