# Supplementary material for: Socioecological Predictors of Child Flourishing and Family Resilience Status Among Children with Adverse Childhood Experiences
Source: Int J Environ Res Public Health. 2026 Feb 24;23(3):277. doi: 10.3390/ijerph23030277 (PMC13026905; doi:10.3390/ijerph23030277)
Supplement: Supplementary file 1 [file ijerph-23-00277-s001.zip › Supplemental Table 2.docx]

Supplemental Table 2. Predicted Probabilities of Child Flourishing and Family Resilience Status from Multinomial Logistic Regression- Full Model (N = 13,571)

|  | Neither Child Flourishing nor Family Resilience | | Child Flourishing Only | | Family Resilience Only | | Child Flourishing and Family Resilience | |
| --- | --- | --- | --- | --- | --- | --- | --- | --- |
|  | AME | SE | AME | SE | AME | SE | AME | SE |
| **Individual Predictors** |  |  |  |  |  |  |  |  |
| Sex (Male = 1) | 0.01 | 0.01 | -0.01 | 0.01 | 0.06*** | 0.01 | -0.06*** | 0.02 |
| Race/Ethnicity (reference group = White, Non-Hispanic) |  |  |  |  |  |  |  |  |
| Hispanic | -0.01 | 0.01 | 0.00 | 0.01 | -0.08*** | 0.02 | 0.08*** | 0.02 |
| Black, Non-Hispanic | -0.01 | 0.02 | 0.01 | 0.01 | -0.07** | 0.02 | 0.08** | 0.02 |
| Other race/ethnicity | 0.00 | 0.01 | 0.03** | 0.01 | -0.05* | 0.02 | 0.01 | 0.02 |
| Child age (years) | 0.00 | 0.00 | 0.00** | 0.00 | -0.01** | 0.00 | 0.00 | 0.00 |
| Access to health care (Yes = 1) | -0.03** | 0.01 | -0.01 | 0.01 | -0.03 | 0.02 | 0.06*** | 0.02 |
| **Parent and Family Predictors** |  |  |  |  |  |  |  |  |
| Parent education (high school or less, reference group = Some college or higher) | 0.01 | 0.01 | 0.00 | 0.01 | -0.05** | 0.02 | 0.04 | 0.02 |
| Family structure (reference group = Two parents, married) |  |  |  |  |  |  |  |  |
| Two parents, not married | 0.03 | 0.02 | -0.02 | 0.02 | -0.04 | 0.03 | 0.03 | 0.03 |
| Single parent | -0.01 | 0.01 | 0.01 | 0.01 | -0.07*** | 0.02 | 0.07*** | 0.02 |
| Other family types | -0.05* | 0.02 | -0.01 | 0.02 | -0.04 | 0.03 | 0.10** | 0.03 |
| Household income (reference group = 400% FPL or higher) |  |  |  |  |  |  |  |  |
| 0-99% FPL | -0.01 | 0.02 | 0.01 | 0.02 | 0.02 | 0.03 | -0.02 | 0.03 |
| 100-199% FPL | -0.01 | 0.02 | 0.02 | 0.01 | -0.03 | 0.02 | 0.02 | 0.02 |
| 200-399% FPL | 0.00 | 0.01 | -0.01 | 0.01 | -0.02 | 0.02 | 0.02 | 0.02 |
| Parenting stress (Yes = 1) | 0.18*** | 0.01 | 0.01 | 0.02 | 0.30*** | 0.03 | -0.49*** | 0.05 |
| **School and Community Predictors** |  |  |  |  |  |  |  |  |
| School safety (Yes = 1) | -0.04* | 0.02 | -0.02 | 0.02 | -0.06 | 0.03 | 0.11** | 0.04 |
| Neighborhood safety (Yes = 1) | -0.00 | 0.02 | -0.01 | 0.02 | 0.03 | 0.03 | -0.02 | 0.04 |
| Supportive neighborhood (Yes = 1) | -0.09*** | 0.01 | -0.02 | 0.01 | -0.05** | 0.01 | 0.15*** | 0.02 |
| Neighborhood amenities (Yes = 1) | -0.02 | 0.01 | -0.01 | 0.01 | -0.00 | 0.01 | 0.03* | 0.02 |
| Distracting neighborhood conditions (Yes = 1) | 0.02 | 0.01 | 0.01 | 0.01 | -0.04 | 0.02 | 0.01 | 0.02 |
| **Cumulative ACEs (reference group = 1 ACE)** |  |  |  |  |  |  |  |  |
| 2 ACEs | 0.03* | 0.01 | 0.01 | 0.01 | 0.05* | 0.02 | -0.08*** | 0.02 |
| 3 ACEs | 0.06* | 0.02 | 0.01 | 0.02 | 0.08** | 0.03 | -0.15*** | 0.03 |
| 4 or more ACEs | 0.09*** | 0.02 | -0.01 | 0.01 | 0.12*** | 0.02 | -0.19*** | 0.02 |

AME = Average Marginal Effect; SE = Standard Error. * p < .05; ** p < .01; *** p < .001.
